# The Abnormal Expression of MicroRNA-542-3p in Hepatocellular Carcinoma and Its Clinical Significance

**DOI:** 10.1155/2018/3973250

**Published:** 2018-02-11

**Authors:** Xi Chen, Qi Zhang, Weijie Ma, Tian Lan, Zhenfei Hong, Yufeng Yuan

**Affiliations:** ^1^Department of Hepatobiliary and Pancreatic Surgery, Zhongnan Hospital of Wuhan University, Wuhan 430071, China; ^2^Department of Gastroenterology, Zhongnan Hospital of Wuhan University, Wuhan 430071, China

## Abstract

**Aim:**

To evaluate the expression of miRNA-542-3p in hepatocellular carcinoma, establish its function, and evaluate whether it could serve as a biomarker for diagnosis and prognosis of HCC patients.

**Methods:**

qRT-PCR analysis was performed to determine the expression level of miRNA-542-3p in normal liver cells and HCC cell lines. Additionally, samples from TCGA consortium and from our patients were analyzed using biostatistical methods to ascertain whether miR-542-3p could be a good biomarker for HCC diagnosis and prognosis. The effects of miRNA-542-3p on HCC were investigated in HCCLM9 cells.

**Results:**

The expression of miRNA-542-3p in HCC cells was significantly downregulated compared with normal liver cells. A lower level of expression of miRNA-542-3p was found in HCC tissue samples than in adjacent normal liver tissue samples from TCGA cases and our patients. Further evaluation revealed that the downregulation was clearly related to aggressive clinicopathological characteristics and affected the prognosis, as low-expressing patients tended to have shorter overall survival. Moreover, cell assays revealed that miR-542-3p overexpression inhibited HCC cell growth and induced apoptosis.

**Conclusion:**

We demonstrated for the first time that miRNA-542-3p appears to function as a novel tumor suppressor in HCC and may serve as a promising prognostic biomarker in HCC patients.

## 1. Introduction

Hepatocellular carcinoma (HCC) accounts for the second highest number of cancer-related deaths worldwide [1]. Although great progress has been made in the research of therapies for HCC, HCC is still a malignant disease with poor prognosis, high rate of recurrence, and metastasis [2]. Usually, patients with HCC have no clinical symptoms during early stages of the disease; therefore, early diagnosis is difficult. Most HCC patients with liver cirrhosis are diagnosed with the disease primarily on the middle-late stages, leading to poor efficacy of the treatment and poor prognosis. Accordingly, early detection and treatment are of great importance. As a simple and effective method, the quantitative determination of serum alpha-fetoprotein (AFP) has become an important test for HCC diagnosis, curative evaluation, and prognosis [3]. However, the quantitative determination of AFP cannot adequately meet the needs for early clinical diagnosis of HCC because the sensitivity and specificity of the AFP test are not accurate enough [4]. Thus, the search for more effective biomarkers for HCC diagnosis has become an urgent endeavor.

MicroRNAs (miRNAs), a group of small noncoding RNAs with a length of about 18~25 nt, are recognized as key regulators of gene expression through their interaction with the 3'-URT of target mRNAs to inhibit their translation or induce their degradation [5, 6]. Abundant evidence suggested that miRNAs play an important role in a variety of human diseases, including cancer [7, 8]. Several studies have shown that, when abnormally expressed, miRNAs function as oncogenes or tumor suppressors in tumorigenesis [9, 10]. miRNAs regulate various cellular processes, which were involved in cancer progression and development, including cell proliferation, apoptosis, migration, and invasion [11]. Previous studies have shown that the aberrant expression of miRNAs in lung cancer [12], breast cancer [13], HCC [14], and pancreatic carcinoma [15] was significantly related with the diagnosis and prognosis. Accordingly, miRNAs have become a new tool for cancer diagnosis, prognosis, and treatment [16]. As a miRNA, miRNA-542-3p plays the role of tumor suppressor in a variety of malignant tumors, such as posttranscriptional regulation of BIRC5 in bladder cancer [17], targeting the AKT pathway in human astrocytoma [18], targeting the oncogene metadherin in gastric cancer [19], and interfering with S1PR1 in breast cancer [20]. More importantly, researchers have reported that miRNA-542-3p is downregulated in HCC, inhibits its growth, and induces apoptosis in HCC cells by targeting FZD7 and inhibiting the Wnt signaling pathway [21]. However, the relationship between miRNA-542-3p expression and clinical pathological parameters has not been well elucidated. To address this issue, we evaluated the expression level of miRNA-542-3p in a normal liver cell line, namely, L-02, and HCC cell lines. In addition, we analyzed the miRNA-542-3p expression level in 53 paired HCC tissue samples and adjacent normal liver tissues from TCGA cases and their relationship with clinical parameters. We also verified the conclusion by analyzing 72 paired HCC tissue samples and corresponding paired adjacent normal liver tissue samples after surgical resection. Additionally, cell proliferation assay, colony formation, cell cycle assays, and cell apoptosis assay were conducted and revealed that miR-542-3p overexpression inhibits HCC cell growth and induces apoptosis. Thus, for the first time, we determined that miRNA-542-3p was associated with the degree of malignancy of the HCC and might be a good candidate biomarker for diagnosis and prognosis of HCC patients.

## 2. Materials and Methods

### 2.1. Clinical Specimens

We initially retrieved the miRNA-543-3p expression data that correlated with clinical parameters of HCC from The Cancer Genome Atlas (TCGA) consortium, and a total of 378 patients were included. Then, cases without miRNA-542-3p status in the tumor tissue or adjacent normal tissue were excluded. Ultimately, 53 patients were included in our analysis of the data from TCGA. In addition, 72 patients (59 men and 13 women) diagnosed with HCC, who had undergone surgery in the Zhongnan Hospital of Wuhan University from 2014 to 2017 and who had not received preoperative chemotherapy or radiation therapy, were also included and analyzed in this study. Paired tissue specimens (tumor and adjacent normal liver tissues), histologically confirmed by experienced pathologists, were collected after receiving informed consent from HCC patients. Fresh tissue samples were collected within 30 minutes after surgery and stored at −80°C in RNAlater® RNA stabilization solution (Invitrogen, Carlsbad, CA, USA) until used. Tumor staging was defined according to the sixth edition of the tumor-node-metastasis (TNM) classification system published by the International Union Against Cancer.

### 2.2. Cell Lines

The human HCC Hep-3B, LM 9, Hep-G2, Hep-G2.215, Huh 7, LM 3 cell lines, and normal liver cell line L-02 were obtained from the Cell Bank, Type Culture Collection, Chinese Academy of Sciences (CBTCCCAS, Shanghai, China). All the cell lines were cultured in Dulbecco's modified Eagle's medium (DMEM; Gibco, Grand Island, NY, USA), supplemented with 10% fetal bovine serum (FBS; Gibco). All cells were cultured at 37°C in a 5% CO_2_ atmosphere.

### 2.3. RNA Extraction and Reverse Transcription

1 mL TRIzol reagent (Invitrogen) per 100 mg of tissue was added to the sample and homogenized using a homogenizer. Then, add 0.2 mL of chloroform per 1 mL of TRIzol reagent used for lysis and securely cap the tube. Subsequently, centrifuge the sample for 15 minutes at 12,000 ×g at 4°C and transfer the aqueous phase containing the RNA to a new tube containing the same volume of isopropanol. Centrifuge for 10 minutes at 12,000 ×g at 4°C. Eventually, wash the RNA using 75% ethanol and elute it with 40 *μ*L RNase-free water. The concentration and purity of the RNA were determined using a NanoDrop ND2000 instrument (Thermo Scientific, Waltham, MA, USA). 1 *μ*g RNA was reverse transcribed in a final reaction volume of 20 *μ*L using the miRNA cDNA Synthesis Kit (ABM Inc., Richmond, BC, Canada). Reverse transcription conditions were as follows: 42°C, 15 min; 70°C, 10 min.

### 2.4. Quantitative Real-Time Polymerase Chain Reaction (qRT-PCR) Analysis

For qRT-PCR, 2 *μ*L of diluted reverse transcription products was mixed with 10 *μ*L SYBR® Green Realtime PCR Master Mix (Toyobo, Osaka, Japan), 1 *μ*L of forward primer (10 *μ*M), 1 *μ*L reverse primers (10 *μ*M), and 6 *μ*L RNase-free water in a final volume of 20 *μ*L according to the manufacturer's instructions. The reaction was performed on a CFX96TM Real-Time System (Bio-Rad, Hercules, CA, USA), with the following reaction conditions: 45 cycles of 95°C, 20 sec; 56°C, 20 sec; 72°C, 20 sec. The presence of a single peak in the melting curve analysis was used to assess the specificity of the PCR amplification. U-6 was chosen as an endogenous control gene to normalize the miRNA-542-3p levels. The sequences of the primers used are listed in Table 1. All experiments were performed in triplicate, and each data point represented the mean results of the triplicate experiments.

### 2.5. Cell Transfections

miR-542-3p mimics and mimic negative controls (NC) were synthesized by RiboBio Corporation (Guangzhou, China). Transfections were performed with Lipofectamine 2000 (Invitrogen) following the manufacturer's instructions.

### 2.6. Cell Proliferation Assay

The Cell Counting Kit-8 (CCK-8; Dojindo, Kumamoto, Japan) assay was used to determine the level of cell proliferation. Briefly, after 48 h of transfection with the miR-542-3p mimics or NC, cells were seeded at 3–5 × 10^3^ cells per well in 96-well plates containing complete culture medium supplemented with 10% FBS. At each time point, 10 *μ*L of the CCK-8 solution was added to each well, and the cells were incubated for 2 h. Subsequently, the absorbance at 450 nm was measured.

### 2.7. Colony Formation Assay

The transfected cells were seeded into 6-well plates at a density of 1000 cells/well in a culture medium containing 0.3% noble agar and grown for two weeks. The cells were fixed in 4% formaldehyde and stained with 0.1% crystal violet for 20 min. The colonies were counted under a light microscope (Olympus, Tokyo, Japan).

### 2.8. Cell Cycle Analysis

After 48 h of transfection with the miR-542-3p mimics or NC, the cells were harvested and fixed in 70% ethanol at 4°C for 24 h. After fixation, the cells were stained with propidium iodide (PI; Beyotime, China) in staining buffer at 37°C in the dark. The cell cycle profiles were analyzed by flow cytometry at an excitation wavelength of 488 nm using an FC500 flow cytometer (Beckman-Coulter, Brea, CA, USA). All experiments were performed in triplicate, and each data point represented the mean results of the triplicate experiments.

### 2.9. Cell Apoptosis Assay

Cells were digested with trypsin without EDTA (Gibco) and were washed twice with phosphate-buffered saline. The cells were treated with Annexin-V-FITC binding buffer, Annexin-V-FITC (Beyotime, China), and PI for 20 min at room temperature. Cell apoptosis was assessed by flow cytometric analysis using an FC500 flow cytometer (Beckman-Coulter). All experiments were performed in triplicate, and each data point represented the mean results of the triplicate experiments.

### 2.10. Statistical Analysis

Statistical analysis was performed using the SPSS 21.0 software (SPSS, Chicago, IL, USA) and GraphPad Prism 5.0 software (GraphPad Software Inc., La Jolla, CA, USA). The results are reported as the means ± SD. The relative miRNA-542-3p expression of our patients was calculated using the comparative cycle threshold (Ct) method (2^−ΔΔCt^), and TCGA patients were calculated using RPM+1. The differences between groups were tested using Student's *t*-test, and two-sided *P* values were calculated. For survival analysis, we divided patients into a low group and a high group by the cut-off of the median that optimally separated the patients and used the Kaplan-Meier method to generate the survival curves. Statistical significance was assigned at *P* < 0.05 (^∗^), *P* < 0.01 (^∗∗^), or *P* < 0.001 (^∗∗∗^).

## 3. Results

### 3.1. miRNA-542-3p Is Significantly Downregulated in HCC Cells and Tissues

**Figure 1:**
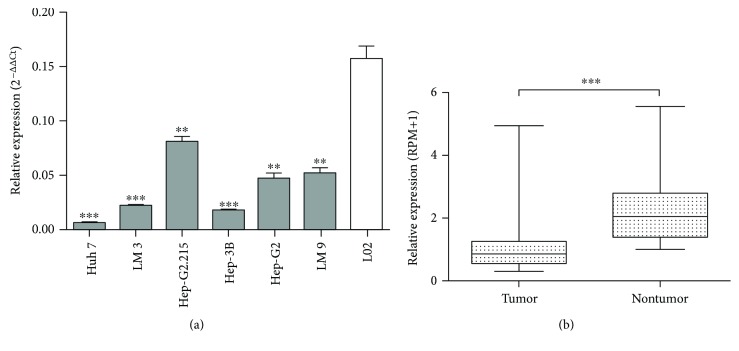
Relative expression level of miRNA-542-3p in HCC cell lines and samples from the dataset of TCGA. (a) miRNA-542-3p expression level was much lower in the six HCC cell lines compared with the normal human liver cell line, L-02. (b) miRNA-542-3p expression level was significantly downregulated in the 53 paired HCC tissues compared with the adjacent normal liver tissues from TCGA. Results are expressed as the mean ± SD. All data were analyzed using Student's *t*-test. ^∗∗^*P* < 0.01, ^∗∗∗^*P* < 0.001.

**Figure 2:**
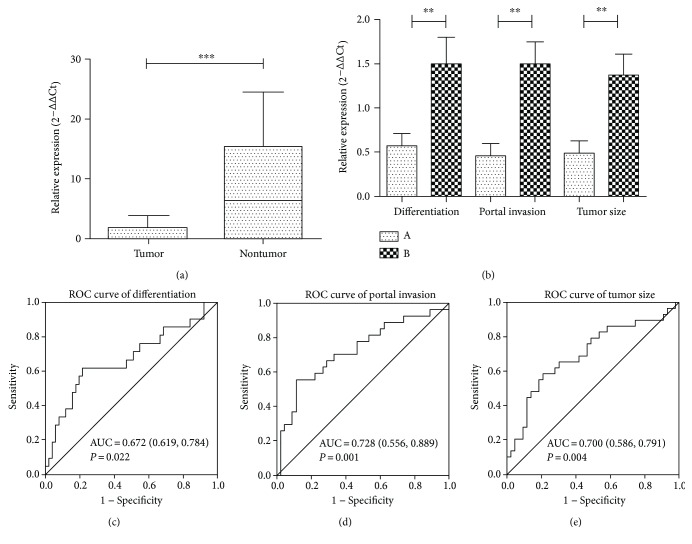
Relative expression level of miRNA-542-3p in HCC tissue and adjacent normal liver tissue and its relationship with clinicopathological parameters. (a) miRNA-542-3p expression level was significantly downregulated in the 72 paired HCC tissues compared with the adjacent normal liver tissues. (b) Highly aggressive tumors tend to have lower miRNA-542-3p expression. Differentiation: A—low/moderate differentiation; B—high differentiation. Portal invasion: A—positive; B—negative. Tumor size: A—≥10 cm; B—<10 cm. (c, d, e) ROC curve analysis of miR-542-3p expression levels for judging aggressive clinicopathologic characteristics. Differentiation: AUC = 0.672 (61.9% sensitivity, 78.4% specificity). Portal invasion: AUC = 0.728 (55.6% sensitivity, 88.9% specificity). Tumor size: AUC = 0.700 (58.6% sensitivity, 79.1% specificity). Results are expressed as mean ± SD. All data were analyzed using Student's *t*-test. ^∗∗^*P* < 0.01, ^∗∗∗^*P* < 0.001.

The expression level of miRNA-542-3p was found to be much lower in the six HCC cell lines examined than in the normal L-02 human liver cell line (Figure 1(a)) (*P* < 0.05). Also, the miRNA-542-3p expression level was significantly downregulated in the 53 paired HCC tissues compared with their corresponding adjacent normal liver tissues (TCGA) (*P* < 0.001), as shown in Figure 1(b). Additionally, our investigation on 72 paired tissues verified this finding (Figure 2(a)) (*P* < 0.001).

### 3.2. The Downregulation of miRNA-542-3p Is Associated with Aggressive Clinicopathological Characteristics of Patients with HCC

According to the analysis of the data from TCGA, the miRNA-542-3p expression was clearly downregulated in patients with portal invasion (Table 2) (*P* = 0.045). Surprisingly, the analysis of the clinical data of our patient-generated results that led to the same conclusion. In addition, we found that expression level of miRNA-542-3p was reduced in patients with moderately lowly differentiated HCC in contrast to that in patients with highly differentiated HCC (*P* = 0.009). Moreover, low miR-542-3p expression was also detected in large tumor volume samples (*P* = 0.004). However, all the evidence indicated that there was no significant difference between miRNA-542-3p expression and other clinical features in HCC, like age, gender, AFP level, cirrhosis, or HBV (Table 3) (*P* ≥ 0.05), although advanced analysis revealed that the expression level of miRNA-542-3p was clearly associated with tumor differentiation (*P* = 0.009), tumor size (*P* = 0.004), and portal invasion (*P* = 0.001) (Figure 2(b)). Additionally, we divided patients into two groups according to the degree of differentiation, portal invasion, and tumor size, and then the receiver operating characteristic (ROC) curves were plotted. The results further proved that miRNA-542-3p strongly influenced the aggressive clinicopathological characteristics of HCC, like tumor differentiation, size, and portal invasion (Figures 2(c)–2(e)).

### 3.3. Low miRNA-542-3p Expression Could Predict Poor Prognosis in HCC Patients

**Figure 3:**
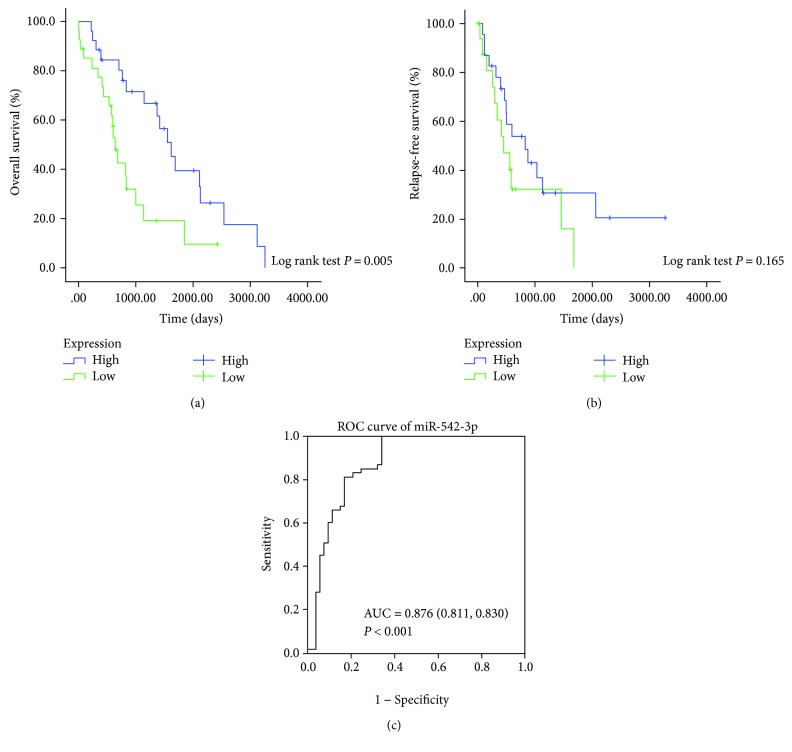
Kaplan-Meier survival curve analysis of miR-542-3p and its ROC curve. (a) Overall survival (OS). (b) Relapse-free survival (RFS) (*P* = 0.165). (c) ROC curve of miR-542-3p for distinguishing HCC tissues from adjacent nontumor tissues. AUC = 0.876 (81.8% sensitivity, 83.0% specificity).

We divided the patients from TCGA datasets into low-/high-expression groups by the cut-off of the median expression value. The results of the Kaplan-Meier survival curve analysis indicated that patients with low miRNA-542-3p expression tended to have shorter overall survival rate than those with high expression. However, the relapse-free survival showed no significant difference between patients with inverse expression status (Figures 3(a) and 3(b)).

### 3.4. Diagnostic Value Analysis of miRNA-542-3p

ROC curve analysis of the data from TCGA was applied to analyze the diagnostic value of miRNA-542-3p. The area under the ROC curve (AUC) of miRNA-542-3p for diagnosing HCC was about 0.876 (81.1% sensitivity, 83.0% specificity) (Figure 3(c)). These findings indicated that miRNA-542-3p expression could be a good candidate to discriminate HCC tissues from normal tissues and ultimately to distinguish patients with HCC from the healthy population.

### 3.5. miRNA-542-3p Inhibits the Proliferation and Induces Apoptosis of HCC Cells

**Figure 4:**
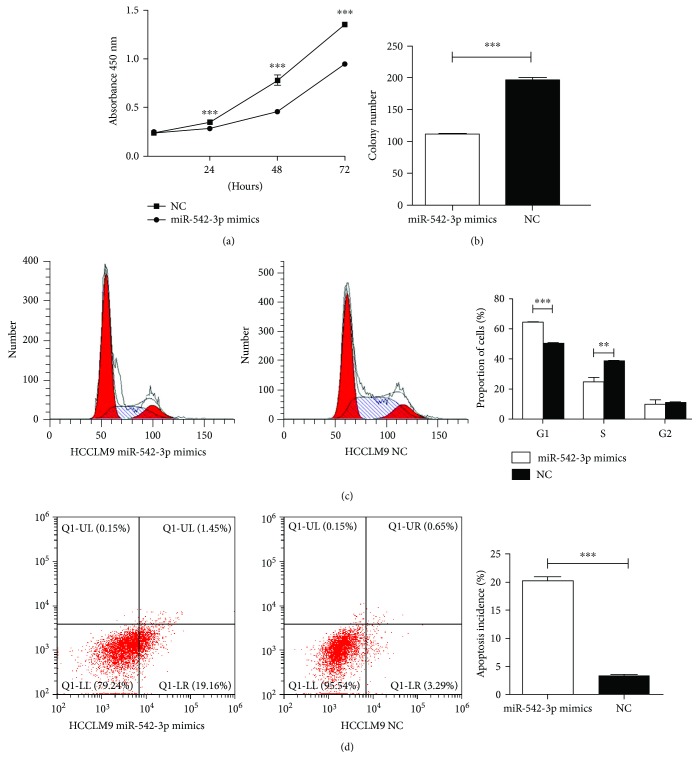
Effect of miR-542-3p overexpression on HCCLM9 cells. (a, b) CCK-8 assay and colony formation assay showed that overexpression of miR-542-3p inhibited proliferation of HCCLM9 cells. (c) Cell cycle analysis showed significant decreases or increases in the proportion of cells in S or G-1 phase, respectively, when miRNA-542-3p was overexpressed in HCCLM9. (d) Apoptosis plot showed that miR-542-3p induced apoptosis in HCCLM9 cells. ^∗∗^*P* < 0.01, ^∗∗∗^*P* < 0.001.

To ascertain the biological role of miR-542-3p in HCC cells, we performed overexpressing experiments by transfecting miR-542-3p mimics and NC into HCCLM9 cell. We then evaluated the effects of miR-542-3p on HCC cell growth using the CCK-8 assay. The results revealed that cell proliferation was significantly inhibited in the cell lines overexpressing miR-542-3p (Figure 4(a)) (*P* < 0.001). To confirm the inhibitory effects of miR-542-3p on HCC cell growth, we performed a colony formation assay. The colony-forming ability of HCCLM9 cells was markedly inhibited by the miR-542-3p overexpression (Figure 4(b)). Furthermore, cell cycle analysis showed significant decreases or increases in the proportion of cells in S or G-1 phase, respectively, when miRNA-542-3p was overexpressed in HCCLM9 (Figure 4(c)). Apoptosis plot showed that miR-542-3p induced apoptosis in HCC-LM9 cells (Figure 4(d)).

## 4. Discussion

In 2002, Calin et al. discovered for the first time that miRNAs are associated with cancer; miRNA15 and miRNA16 were found to be downregulated in chronic lymphocytic leukemia [22]. Since then, more and more miRNAs have been studied to determine their relationship with cancer. Indeed, a large number of studies have revealed that the abnormal expression of miRNAs is a common occurrence in malignant tumors [23]. For example, the upregulation of miRNA-155 was found to be significantly connected with a worse prognosis in lung cancer [12]. Moreover, miRNAs were also associated with many other diseases, like metabolic disorders [24]. Also, the upregulation of miRNA-19 [25], miRNA-21 [26], and miRNA-221/222 [27] was reported to regulate drug resistance of tumor. The present studies suggest that miRNAs might act as oncogene by targeting tumor suppressor genes, like miRNA-372, for instance, which can suppress LATS2 and induce testicular germ cell tumors [28]. Previous studies also confirmed that they exert their antitumor effects by affecting oncogenes, for instance, let-7 which negatively controls the oncogene RAS [29]. Negrini et al. [30] found that miRNA-122, miRNA-199, miRNA-221, and miRNA-21 can be used in the diagnosis and prognosis of patients with HCC through influencing the expression of genes by targeting different signaling pathway. As a miRNAs, miRNA-542-3p has attracted increasing attention from researchers; its tumor suppression function was convincingly shown in many studies. Wu et al. [21] have demonstrated that miRNA-542-3p inhibits the growth of HCC cells by targeting the FZD7/Wnt signaling pathway, but the clinical significance of miRNA-542-3p in HCC is not yet well understood.

## 5. Conclusion

Overall, our studies established the relation between the expression of miRNA-542-3p and clinicopathological parameters of HCC. miRNA-542-3p expression level was lower in the HCC tissues than in the corresponding noncancerous tissues, and clinicopathological parameters like tumor differentiation, portal invasion, and tumor size were closely related to its expression. Low miRNA-542-3p led to poor prognosis; it increased the probability of a malignant phenotype and worse prognostic phenotype. Accordingly, we confirmed that miRNA-542-3p can act as a prognostic biomarker of HCC. Furthermore, our results from various in vitro experiments supported a tumor suppressor role for miRNA-542-3p in HCC. Moreover, our study provided the first evidence indicating that the miRNA-542-3p expression is closely related to HCC clinical features. Additionally, we also confirmed that the miRNA-542-3p can be a good biomarker to diagnose HCC. Nevertheless, although important findings were revealed by our study, the specific mechanistic roles played by miRNA-542-3p in the development of HCC have not been clarified. Future studies should focus on deciphering the specific functions of miRNA-542-3p in more detail to help identify effective therapeutic targets.

## Figures and Tables

**Table 1:** Sequences of primers.

Name	Forward	Reverse
miR-542-3p	TGTGACAGATTGATAACTGAAA	GTGCAGGGTCCGAGGT
RNA U6	GCTTCGGCAGCACATATACTAAAAT	CGCTTCACGAATTTGCGTGTCAT

**Table 2:** Association of miRNA-542-3p expression with clinicopathological parameters in HCC (TCGA).

Clinical parameters	*n*	miRNA-542-3p relative expression (2^−ΔΔCt^)		
		Mean ± SD	t	*P*
Age				
<50	8	0.719 ± 0.407	1.273	0.222
≥ 50	45	1.133 ± 0.926		
Gender				
Male	30	0.883 ± 0.472	−1.6408	0.113
Female	23	1.314 ± 1.190		
AFP (ng/mL)				
<400	30	1.080 ± 0.843	−0.496	0.623
≥ 400	7	0.917 ± 0.379		
Missing data	16			
Cirrhosis				
Negative	25	0.950 ± 0.529	0.782	0.439
Positive	13	1.088 ± 0.500		
Missing data	15			
TNM stage				
I-II	29	0.872 ± 0.487	1.469	0.149
III-IV	15	1.228 ± 1.1253		
Missing data	9			
HBV				
Negative	31	1.101 ± 1.057	−0.303	0.763
Positive	22	1.027 ± 0.553		
Portal invasion^∗^				
Negative	32	1.295 ± 1.039	−2.059	0.045^∗^
Positive	15	0.721 ± 0.390		
Missing data	6			

Data are presented as mean ± SD. ^∗^*P* < 0.05.

**Table 3:** Association of miRNA-542-3p expression with clinicopathological parameters in HCC.

Clinical parameters	*n*	miRNA-542-3p relative expression (2^−ΔΔCt^)		
		Mean ± SD	*t*	*P*
Age				
<50	24	0.829 ± 1.200	0.048	0.961
≥50	48	0.843 ± 1.213		
Gender				
Male	59	0.907 ± 1.218	1.026	0.308
Female	13	0.530 ± 1.101		
AFP (ng/mL)				
<400	33	0.854 ± 1.264	−0.102	0.919
≥400	39	0.825 ± 1.158		
Cirrhosis				
Negative	28	1.065 ± 1.289	−1.248	0.218
Positive	44	0.694 ± 1.130		
Size (cm)^∗^				
<10	29	1.362 ± 1.330	−3.047	0.004^∗∗^
≥10	43	0.485 ± 0.967		
Differentiation^∗∗^				
High	21	1.497 ± 1.379	−2.794	0.009^∗∗^
Low/moderate	51	0.567 ± 1.012		
TNM stage				
I-II	28	0.582 ± 1.056	1.459	0.149
III-IV	44	1.002 ± 1.267		
HBV				
Negative	25	0.623 ± 1.039	1.187	0.240
Positive	47	0.953 ± 1.272		
Portal invasion^∗^				
Negative	27	1.483 ± 1.351	−3.514	0.001^∗^
Positive	45	0.452 ± 0.914		

Data are presented as mean ± SD. ^∗^*P* < 0.05. ^∗∗^*P* < 0.01.

## References

[1] Siegel R. L., Miller K. D., Jemal A. (2016). Cancer statistics, 2016. *CA: A Cancer Journal for Clinicians*.

[2] Mazzoccoli G., Tarquini R., Valoriani A., Oben J., Vinciguerra M., Marra F. (2016). Management strategies for hepatocellular carcinoma: old certainties and new realities. *Clinical and Experimental Medicine*.

[3] Faivre S., Bouattour M., Raymond E. (2011). Novel molecular therapies in hepatocellular carcinoma. *Liver International*.

[4] Yuen M. F., Hou J. L., Chutaputti A., Asia Pacific Working Party on Prevention of Hepatocellular Carcinoma (2009). Hepatocellular carcinoma in the Asia pacific region. *Journal of Gastroenterology and Hepatology*.

[5] Bartel D. P. (2004). MicroRNAs: genomics, biogenesis, mechanism, and function. *Cell*.

[6] Winter J., Jung S., Keller S., Gregory R. I., Diederichs S. (2009). Many roads to maturity: microRNA biogenesis pathways and their regulation. *Nature Cell Biology*.

[7] Lu J., Getz G., Miska E. A. (2005). MicroRNA expression profiles classify human cancers. *Nature*.

[8] Baranwal S., Alahari S. K. (2010). miRNA control of tumor cell invasion and metastasis. *International Journal of Cancer*.

[9] Schickel R., Boyerinas B., Park S. M., Peter M. E. (2008). MicroRNAs: key players in the immune system, differentiation, tumorigenesis and cell death. *Oncogene*.

[10] Tutar L., Tutar E., Tutar Y. (2014). MicroRNAs and cancer; an overview. *Current Pharmaceutical Biotechnology*.

[11] Lin S., Gregory R. I. (2015). MicroRNA biogenesis pathways in cancer. *Nature Reviews Cancer*.

[12] Yanaihara N., Caplen N., Bowman E. (2006). Unique microRNA molecular profiles in lung cancer diagnosis and prognosis. *Cancer Cell*.

[13] Sempere L. F., Christensen M., Silahtaroglu A. (2007). Altered MicroRNA expression confined to specific epithelial cell subpopulations in breast cancer. *Cancer Research*.

[14] Hayes C. N., Chayama K. (2016). MicroRNAs as biomarkers for liver disease and hepatocellular carcinoma. *International Journal of Molecular Sciences*.

[15] Papaconstantinou I. G., Manta A., Gazouli M. (2013). Expression of microRNAs in patients with pancreatic cancer and its prognostic significance. *Pancreas*.

[16] Hayes J., Peruzzi P. P., Lawler S. (2014). MicroRNAs in cancer: biomarkers, functions and therapy. *Trends in Molecular Medicine*.

[17] Zhang J., Wang S., Han F. (2016). MicroRNA-542-3p suppresses cellular proliferation of bladder cancer cells through post-transcriptionally regulating survivin. *Gene*.

[18] Cai J., Zhao J. J., Zhang N. (2015). MicroRNA-542-3p suppresses tumor cell invasion via targeting AKT pathway in human astrocytoma. *The Journal of Biological Chemistry*.

[19] Shen X., Si Y., Yang Z., Wang Q., Yuan J., Zhang X. (2015). MicroRNA-542-3p suppresses cell growth of gastric cancer cells via targeting oncogene astrocyte-elevated gene-1. *Medical Oncology*.

[20] Wu H. X., Wang G. M., Lu X., Zhang L. (2017). miR-542-3p targets sphingosine-1-phosphate receptor 1 and regulates cell proliferation and invasion of breast cancer cells. *European Review for Medical and Pharmacological Sciences*.

[21] Wu W., Dang S., Feng Q., Liang J., Wang Y., Fan N. (2017). MicroRNA-542-3p inhibits the growth of hepatocellular carcinoma cells by targeting FZD7/Wnt signaling pathway. *Biochemical and Biophysical Research Communications*.

[22] Calin G. A., Dumitru C. D., Shimizu M. (2002). Frequent deletions and down-regulation of micro- RNA genes *miR15* and *miR16* at 13q14 in chronic lymphocytic leukemia. *Proceedings of the National Academy of Sciences of the United States of America*.

[23] Croce C. M. (2009). Causes and consequences of microRNA dysregulation in cancer. *Nature Reviews Genetics*.

[24] Poy M. N., Eliasson L., Krutzfeldt J. (2004). A pancreatic islet-specific microRNA regulates insulin secretion. *Nature*.

[25] Liang Z., Li Y., Huang K., Wagar N., Shim H. (2011). Regulation of miR-19 to breast cancer chemoresistance through targeting PTEN. *Pharmaceutical Research*.

[26] Shi G. H., Ye D. W., Yao X. D. (2010). Involvement of microRNA-21 in mediating chemo-resistance to docetaxel in androgen-independent prostate cancer PC3 cells. *Acta Pharmacologica Sinica*.

[27] Garofalo M., Di Leva G., Romano G. (2009). *miR-221&222* regulate TRAIL resistance and enhance tumorigenicity through PTEN and TIMP3 downregulation. *Cancer Cell*.

[28] Voorhoeve P. M., le Sage C., Schrier M. (2006). A genetic screen implicates miRNA-372 and miRNA-373 as oncogenes in testicular germ cell tumors. *Cell*.

[29] Johnson S. M., Grosshans H., Shingara J. (2005). *RAS* is regulated by the let-*7 microRNA* family. *Cell*.

[30] Negrini M., Gramantieri L., Sabbioni S., M. Croce C. (2011). microRNA involvement in hepatocellular carcinoma. *Anti-Cancer Agents in Medicinal Chemistry*.

